# Factors that influence adolescent girls and young women's re‐initiation or complete discontinuation from daily oral PrEP use: a qualitative study from Eastern Cape Province, South Africa

**DOI:** 10.1002/jia2.26175

**Published:** 2023-09-27

**Authors:** Lindsey de Vos, Emily Krogstad Mudzingwa, Lauren Fynn, Millicent Atujuna, Matinatsa Mugore, Monica Gandhi, Connie Celum, Sybil Hosek, Linda‐Gail Bekker, Joseph Daniels, Andrew Medina‐Marino

**Affiliations:** ^1^ Research Unit Foundation for Professional Development East London South Africa; ^2^ The Desmond Tutu HIV Centre University of Cape Town Cape Town South Africa; ^3^ Institute for Collaboration on Health, Intervention, and Policy University of Connecticut Storrs Connecticut USA; ^4^ Mailman School of Public Health Columbia University New York New York USA; ^5^ Division of HIV, Infectious Diseases and Global Medicine, Department of Medicine University of California, San Francisco San Francisco California USA; ^6^ Departments of Global Health, Medicine, and Epidemiology University of Washington Seattle Washington USA; ^7^ Division of Infectious Diseases Stroger Hospital of Cook County Chicago Illinois USA; ^8^ Division of Child and Adolescent Psychiatry Department of Psychiatry Stroger Hospital of Cook County Chicago Illinois USA; ^9^ Edson College of Nursing and Health Innovation Arizona State University Phoenix Arizona USA; ^10^ Perelman School of Medicine University of Pennsylvania Philadelphia Pennsylvania USA

**Keywords:** oral pre‐exposure prophylaxis, PrEP, adherence, discontinuation, adolescent girls and young women, South Africa

## Abstract

**Introduction:**

Adolescent girls and young women (AGYW) face barriers that jeopardize their prevention‐effective use of daily oral pre‐exposure prophylaxis (PrEP). We sought to understand factors that influence AGYW's prolonged breaks in PrEP use, and their decisions to re‐initiate or discontinue using PrEP in the context of a community‐based adherence support intervention.

**Methods:**

In‐depth interviews (IDIs) were conducted between December 2019 and April 2021 with purposively selected AGYW (aged 16–25) enrolled in the Community PrEP Study (CPS) in Buffalo City Metro Health District, Eastern Cape Province, South Africa. AGYW were offered monthly PrEP for 24 months at two community‐based study sites. Interview guides were informed by the Information‐Motivation‐Behavioural Skills Model, and data were analysed using illustrative code reports and a case analysis.

**Results:**

A total of 603 participants were enrolled and initiated on PrEP in the parent study. Fifty‐three IDIs were conducted with 50 CPS participants. Findings revealed that external factors (e.g. local movement, school holidays and medication side‐effects) and social conflicts (e.g. discretion and partner mistrust) directly influenced breaks in PrEP usage. A decrease in one's self‐perception of HIV risk prolonged the duration of these “PrEP breaks.” Once PrEP refill visits were missed, some AGYW delayed returning for refills out of fear of being scolded by study staff. The differences between those participants who eventually re‐initiated PrEP and those who disengaged from PrEP use can be attributed to social support and encouragement, level of familiarity with PrEP, risk perceptions, self‐initiated discussions with staff and diminishing side effects.

**Conclusions:**

Despite implementing a community‐based PrEP delivery platform and behavioural intervention that included support for daily oral PrEP adherence and disclosure, participants struggled with consistent daily oral PrEP use. Unpredictable life events, including local movement and schooling schedules, in addition to being judged for their perceived behaviours, pose a challenge for consistent pill pick‐up for AGYW and habit formation. Long‐acting injectable PrEP may mitigate a number of these external barriers. Interventions that integrate long‐term planning skills, how to navigate existing social judgements and how to access sources of social support may further improve habit formation for PrEP use, regardless of its formulation.

## 1 INTRODUCTION

In 2022, 7.6 million adults and children were living with HIV in South Africa, with the prevalence among women aged 15–49 years estimated to be nearly double that of men (24.3% vs. 12.5%) [1, 2, 3]. Incidence rates among adolescent girls and young women (AGYW) aged 15–24 years is three times higher than adolescent boys and young men (1.51% vs. 0.49%, respectively) [4]. Contributing to AGYW's disproportionate vulnerability to HIV are biological, social and structural factors, including gender‐based violence, gender inequities, socio‐economic burdens and HIV‐transmission networks involving age‐disparate sexual partners [5, 6, 7].

Daily oral pre‐exposure prophylaxis (PrEP) for HIV prevention is highly effective when adherence to treatment regimens is consistently high (i.e. taken in a prevention‐effective manner) [8, 9]. This means that AGYW take at least >4 daily oral PrEP tablets a week to have protection during periods of risk [10]. Unfortunately, numerous clinical studies have reported inconsistent or discontinuation of PrEP use, with only 25%–50% of AGYW showing high drug levels (tenofovir diphosphate [TFV‐DP] ≥700 fmol/dried blood spot punch [DBS]) after the first 3 months [11, 12, 13, 14, 15, 16, 17, 18, 19, 20]. This dramatically minimizes its prevention‐effective benefits (i.e. protective coverage of unprotected sex acts), and reveals gaps in the PrEP care cascade. Although it has been argued that PrEP is under the control of its end‐users, psychosocial, logistic and structural barriers outside their control impact users ability to effectively use and continue with daily oral PrEP [13, 14, 19, 21–28]. These include HIV‐related stigma [13, 14, 19, 21, 22], social support [23–26, 29], relational conflicts [26–28, 30–32], adaptive use or seasons of risk [23, 28, 30, 33, 34], sexuality stigma [35, 36, 37], and structural and health system issues for PrEP pick‐up [24, 27, 28, 35, 38, 39].

Given these barriers, the high burden of HIV among AGYW in South Africa, and the growing importance of delivering health services in the community, the Community PrEP Study (CPS) was implemented to assess the acceptability, feasibility and impact of community‐based platforms to increase access, uptake and prevention‐effective use of PrEP [40]. To inform the development of future adherence support programmes, we conducted a qualitative sub‐study to understand factors that continue to influence AGYW's PrEP use. We designed and implemented a behavioural intervention aimed at maximizing prevention‐effective use of PrEP by focusing on determining what influences prolonged breaks in PrEP use and the decision to re‐initiate or discontinue using PrEP [30, 41].

## 2 METHODS

### 2.1 Overview of CPS

CPS has been described previously [40, 42]. Briefly, CPS leveraged community‐based HIV testing (CBCT) platforms to increase AGYW's access and uptake to HIV PrEP services. It further implemented and evaluated behavioural intervention programmes that included adherence support activities to improve AGYW's prevention‐effective use of PrEP [40]. Given AGYW's high risk for HIV acquisition in South Africa, those who met the following criteria were eligible for PrEP initiation during the CPS: 16–25 years old, confirmed HIV‐negative through CBCT, self‐identified as female, not currently pregnant or breastfeeding, not participating in another HIV prevention study or using PrEP, planning to reside within the study area for the next 12 months and provision of informed consent. The current behavioural intervention delivered by lay‐health counsellors as either a group‐based or one‐on‐one programme, included adherence support counselling and the development of action plans for problem‐solving. Adherence was measured using TFV‐DP concentrations in DBS samples, defining high and low adherence as ≥700 versus ≤699 fmol/DBS punch [37] collected at baseline and at specific study follow‐up visits [40].

### 2.2 Study setting

CPS was conducted in two communities (one rural, one peri‐urban) in the Buffalo City Metro (BCM) Health District, Eastern Cape Province, South Africa. The Eastern Cape has an estimated HIV prevalence of 12.3% (95% CI: 8.2–18.1) among youth aged 15–24 years and an incidence of 1.91% among AGYW 16–25 years [4, 43]. Furthermore, an estimated 15.6% of youth (aged 15–24 years) in the Eastern Cape reported early sexual debut (i.e. before age 15) [4]. The BCM health district has a population of ∼800,000, an estimated population density of 298 persons per km^2^, a youth unemployment rate of ∼45% and a secondary education completion rate of 27.2% [44]. Most residents speak isiXhosa, and approximately 25% of the population in the BCM reside in informal housing [45].

### 2.3 Participant recruitment and data collection

Participants enrolled in CPS who met interview category case definitions (Supporting Information, Table S1) and who were willing to participate in an in‐depth interview (IDI) were eligible for inclusion. Eligible participants were contacted by field staff and received R100 (∼$6.50 USD) and a snack to compensate participants for their time and travel costs. Participants were interviewed between 1 December 2019 and 30 April 2021. Separate semi‐structured interview guides were developed by MA, AMM, LF and MM for each interview category (Supporting Information, Table S1), and are available in both English and IsiXhosa depending on a participant's preferred language. IDI guide development was informed by the Information‐Motivation‐Behavioural Skills Model of Behaviour Change (IMB), which incorporates four critical components for HIV‐related health behaviours: (1) accurate information; (2) motivation; (3) self‐efficacy; and (4) behavioural skills [46, 47]. Although IDI guides developed for each interview category had different key areas of inquiry, a number of domains remained constant across interview categories, including: (1) PrEP initiation and motivation; (2) individual PrEP use and experience narratives; (3) disclosure events; (4) perceived risk; (5) PrEP pick‐up challenges; (6) disrupted use and adherence barriers; (7) perceptions of and experiences with study site and staff; (8) PrEP preferences; and (9) recommendations to improve PrEP pick‐up and support for other AGYW.

All participants were interviewed by female research staff. Interviews were conducted face‐to‐face in a private space at community study sites. All study staff were trained on the study protocol and in the conduct of human subject research, including confidentiality protections. Staff also received a 3‐day training on qualitative research, interviewing skills and interview guides, with refresher training sessions provided as each IDI guide was deployed. IDIs lasted an average of 25 minutes, and were audio‐recorded, transcribed and translated into English (if performed in isiXhosa). Transcripts were reviewed by a second member of the qualitative research team (QRT) for quality assurance. QRT members held regular study meetings to discuss and refine data collection and analysis processes.

### 2.4 Data analysis

The study team developed an iteratively refined codebook and identified key codes through discussions of interview transcripts, emerging themes and study objectives. The codebook was applied to all transcripts independently, reviewed by a second QRT member for inter‐coder reliability and analysed using Dedoose software (v8.3.43, Los Angeles, CA: SocioCultural Research Consultants, LLC). Discrepancies were addressed during weekly meetings. We exported and analysed specific code reports with illustrative excerpts that described: (1) short, mid‐ and long‐term breaks; (2) motivations for PrEP, including missed visits; (3) discontinued use and re‐initiation; (4) inconsistent PrEP pick‐up; and (5) seasons of risk. Code reports were inductively analysed across interview categories (Supporting Information, Table S1). The analysis involved writing summary memos, which included: (1) noted unique observations; (2) comparing participant groups; and 3) theme discussions. Preliminary findings and exemplifying cases relating to reasons for taking PrEP breaks were discussed among the QRT to refine results.

### 2.5 Ethics approval

Ethics approval was granted by the University of Cape Town's Human Research Ethics Committee (Ref no.: 289/2018). The Eastern Cape Provincial Research Committee and the BCM Health District provided permission to conduct the study. Written informed consent was obtained from all interviewed participants in addition to the prior consent to enrol in the CPS parent study.

### 2.6 Participant representation

In‐text quotes are denoted as “age, study community, interview category.” Examples of PrEP breaks and re‐initiation were assessed across all interview categories (Supporting Information, Table S1).

## 3 RESULTS

Six‐hundred and three participants consented and initiated PrEP as part of the parent CPS study, of which 329 (54.6%) presented for a first medication refill within 90 days of accepting their first bottle of PrEP [42]. The average PrEP refill rate for the first 6 months was 48.8%, with declining attendance over time; 60 participants (15.8%) attended their last study visit at month 24 (data not shown). Throughout CPS, a total of 84 participants were withdrawn at their request, while study participation was terminated for 12 participants due to seroconversion (data not shown).

All participants who were successfully contacted for an IDI and presented at the study site provided written informed consent. Fifty‐three IDIs were conducted among 50 participants (multiple IDI eligibility). In Table 1, participant socio‐behavioural characteristics are described; note, not all 50 participants presented at each of the follow‐up study visits where behavioural surveys were administered. Briefly, the median age of interviewed participants was 18.5 years (interquartile range [IQR] = 17–22 years). At the time of recruitment, most participants accessed HIV testing services via pop‐up testing sites (82.0%), 64.0% were currently in school and 62.0% reported ever having had sex.

**Table 1:** Participant characteristics at baseline, 3‐, 12‐ and 24‐month follow‐up (*N* = 50)

**At baseline**		
Age in years (median, IQR)		18.5 (17.0−22.0)
Study site	*Peri‐urban*	32 (64.0%)
	*Rural*	18 (36.0%)
CBCT modality	*Mobile*	41 (82.0%)
	*Household*	9 (18.0%)
In school		32 (64.0%)
No. of people in household (median, IQR)		5 (4−7)
Ever had sexual intercourse	*Yes*	31 (62.0%)
	*No*	15 (30.0%)
	*Refused to answer*	4 (8.0%)
Age of sexual debut (median, IQR)		17 (15−18)
Frequency condom use^a^ (past 6 months)	*All the time*	13 (37.1%)
	*Some of the times*	17 (48.6%)
	*Never*	5 (14.3%)
Multidimensional perceived social support^b^ (median, IQR)		5.6 (5−6.3)

**At M3 (*n* = 29), M12 (*n* = 26) and M24 (*n* = 25)** ^c^		
Sexual intercourse since last interview		
	*Between baseline and month 3*	9 (31.0%)
	*Between month 3 and month 12*	16 (61.5%)
	*Between month 12 and month 24*	17 (68.0%)
Disclosed PrEP use to someone since last interview		
	*Between baseline and month 3*	14 (48.3%)
	*Between month 3 and month 12*	25 (96.2%)
	*Between month 12 and month 24*	20 (80.0%)
Experienced side‐effects since last interview		
	*Between baseline and month 3*	3 (10.3%)
	*Between month 3 and month 12*	4 (15.4%)
	*Between month 12 and month 24*	5 (20.0%)
Perceived HIV risk^d^ (median, IQR)		
	*Between baseline and month 3*	1 (0−2)
	*Between month 3 and month 12*	1.5 (0−3)
	*Between month 12 and month 24*	1 (0−2)

*Note*: Values are (*n*, %) unless otherwise specified.

Abbreviations: CBCT, community‐based HIV counselling and testing; IQR, interquartile range; M3, 3‐month follow‐up; M12, 12‐month follow‐up; M24, 24‐month follow‐up.

^a^ *n* = 35 participants who responded ever having had sexual intercourse or initially did not respond.

^b^ Total median score for perceived social support (1 = low support; 7 = high support) as sourced from significant others, family members or friends.

^c^ Administered among those who visited during their month 3, month 12 or month 24 study visit (pertaining to the past 3, 9 or 12 months between these study visits).

^d^ Based on their own behaviour, participants were asked to rate their HIV risk on a scale of 0 to 10.

**Figure 1:**
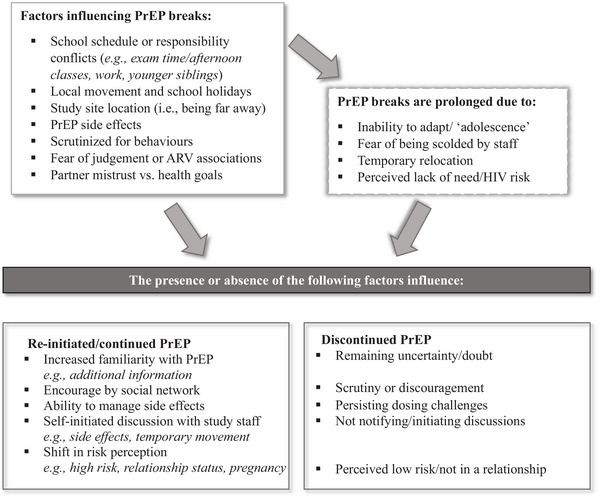
Summary of qualitative findings. Short or long PrEP breaks, and the presence or absence of certain factors that determine whether someone re‐initiates versus discontinues PrEP.

Reasons for taking breaks from PrEP, and factors that determine prolonged breaks and/or whether someone re‐initiated PrEP are represented in Figure 1. Participants described how their PrEP use was disrupted by conflicting schedules and social scrutiny. The duration of PrEP breaks was prolonged by the inability to adjust to these logistic and social conflicts, perceived lack of need/risk and/or fear of being scolded by study staff. The presence or absence of certain factors determined whether a participant discontinued or re‐initiated PrEP after taking breaks including PrEP familiarity, self‐perceived HIV risk, social encouragement and PrEP side effects.

### 3.1 Local movement, conflicting schedules and behaviour scrutiny disrupt PrEP adherence

#### 3.1.1 Local movement and conflicting schedules

A common perception among study participants as to why other AGYW might miss a dose or picking‐up a PrEP medication refill included local movement, weekend getaways, exam‐writing, socializing and school holidays. Participants gave similar examples when they themselves missed and/or took short breaks from using PrEP. AGYW who described conflicting or disrupted schedules were more likely to also discuss missed doses of PrEP. Such difficulties indicated both an inability to plan long‐term and intention to not take PrEP at certain times.

“A person's routine changes and they won't wake up at the time they usually do when they are going to school and when they are on holiday. They won't take their pills with them because they did not tell other people in the family about PrEP.” (16 years, Rural, Two or more consecutive missed visits)

AGYW explained the difference between PrEP use during school versus when on holiday/in the village. AGYW felt that they generally do not always disclose outside of their usual social network, for example when they are out with friends or visiting extended family, and feared discouragement:

“…When it's festive it's [PrEP] not being taken at all. When you are in the villages no you can't take your pills in the villages cause people there, they'll look at me like I'm sick.” (18 years, Peri‐urban, Sero‐converter)

Participants reported logistical challenges when travelling and not taking PrEP with them on holiday/to the village, and at times preferred not to use PrEP due to anticipated stigma. Unfortunately, these disruptions led to inconsistent PrEP reminders, non‐adherence and missed monthly sessions, posing higher HIV acquisition risks:

“…what am I going to say, it's not condomizing and confusing the time to take this pill…of PrEP. And then maybe, maybe I wouldn't be here and then not able to take them, you see… Sometimes you go to the villages for about an entire month and then not take the pills with you.” (18 years, Peri‐urban, Sero‐converter)

#### 3.1.2 Behaviour scrutiny including partner mistrust

AGYW anticipated judgement and mistrust from others. Participants discussed how PrEP was likened to antiretroviral therapy and examples of judgement were reported from friends, parents and partners:

“she [my friend] criticises that I take it every day. She says it's like I am taking ARVs that's the only thing… I also see what she is talking about, but I wanted to protect myself that time.” (18 years, Peri‐urban, Serial Interview)

“…Then I try to explain [PrEP], and you [as if staff is a friend] will take whatever that suits you and you will discourage me and will see that my friend is telling the truth you see, there is no difference because it's the same as if I'm using ARVs.” (24 years, Rural, Unique pattern of medication use)

Further, a lack of support, social acceptance and partner mistrust may negatively influence the use of PrEP:

“Firstly, some of them [that stop taking PrEP] don't get support from their homes, some they are made to go up and down by… their partners… because others ask questions like, ‘why are you taking PrEP, don't you trust me or are you sleeping with other men?’” (21 years, Peri‐urban, Two or more consecutive missed visits)

Another participant mentioned she did not disclose to her partner as she feared, *“he would overpower me with questions”* (18 years, Peri‐urban, Serial Interviews M18–M24).

AGYW perceived other AGYW to lack negotiating power with partners or parents. As a result, they thought other AGYW using PrEP felt shame or engaged in confrontation avoidance, both of which may disrupt PrEP use:

“…like they [other AGYW] go to their boyfriend for those days and the boyfriend is not comfortable with the fact that she is taking this [PrEP] pill so they don't take it because the boyfriend is against it” (21 years, Peri‐urban, Two or more consecutive missed visits)

‘‘I: What do you think are the reasons why some young women are unable to pick up their pills?

P: Maybe the miscommunication of the partners or not having a trust that these pills are really protecting this virus, or you are lying to your parents. I think so.’’ (19 years, Rural, Unique pattern of medication use)

### 3.2 Factors that prolonged PrEP breaks

AGYW who missed two or more consecutive monthly refill visits described difficulties in adapting PrEP to routine changes. These same AGYW went on to describe the prolongation of their PrEP break resulting from: (1) fear of being scolded by study staff upon their return; (2) a perceived lack of need for PrEP; and (3) not having clearly defined reasons for taking PrEP (e.g. motivated by PrEP goals).

“I was just lazy… like since it has been so long since I last came [to the study site], I thought when I come back, they'll scold me asking me questions like ‘why didn't you come’ but that didn't happen” (20 years, Peri‐urban, Two or more consecutive missed visits)

“Oh, I was scared of being yelled at on why I didn't come to pick up my pills, so I thought I shouldn't come indefinitely… When I came, they [staff] said… you won't be asked anything and they won't scold you for not coming, one can feel free…” (17 years, Peri‐urban, Two or more consecutive missed visits)

These AGYW seemed unsure and feared being interrogated (“asking questions”) by study staff once study visits were missed. However, those who returned expressed not encountering this reaction from study staff members. Participants who were less familiar or confident about PrEP at the time that they took a break were more likely to delay re‐initiation.

“…firstly in December I started a holiday job and I had to work every day so after December then uhm I was scared to come since I was new here and I was not used to the site” (19 years, Peri‐urban, Two or more consecutive missed visits)

Many AGYW discussed the challenges of temporary relocations and the inability to access PrEP outside of the study or defined clinic locations as factors impacting prolonged breaks in PrEP use. Participants also discussed the intermittent use of PrEP by other AGYWs depending on their relationship status, and whether they perceived themselves at risk for HIV. This AGYW gives an example of seasons of risk among other participants, that determine PrEP use: *“The one [another participant] that I am going to talk about, when she doesn't have a boyfriend, she doesn't come to take it. When she does have [a boyfriend], she does come.”* (20 years, Peri‐urban, Two or more consecutive missed visits)

### 3.3 Social networks and self‐perceived risk influence re‐initiation on PrEP

Despite missing two or more continuous months of PrEP refill visits, some AGYW chose to re‐initiate PrEP. Encouragement from mothers, partners and friends, as well as shifts in self‐perceived risk of HIV acquisition, strongly influenced AGYW's decision to re‐initiate PrEP, even among AGYW who were initially unsure about PrEP. Mothers showed support through refill reminders and recognizing the benefits of PrEP. Friends or peers familiar with PrEP also influenced AGYW re‐initiation decisions:

“I am a person that stays at home, it is my mom that keeps saying ‘go to N. [study site] and take your pills that help…’” (20 years, Rural, Two or more consecutive missed visits).

“…yes cause my friend was coming more frequently [to the monthly support visits] so that is what motivated me to come cause she used to tell me how safe she feels now that she uses PrEP” (19 years, Peri‐urban, Two or more consecutive missed visits).

Additionally, many AGYW decided to re‐initiate PrEP due to changes in their self‐perceived risk for HIV acquisition, “It is the ways of the guy that I'm in a relationship with, I see that it is not good for me. Because I think in my mind, I'm not sure that he is cheating on me” (19 years, Rural, Unique patterns) but also because of supportive partners. The following AGYW shows how encouragement from her partner enabled her to re‐initiate PrEP despite prior pick‐up challenges (being away):

“What influenced me to come back after I missed taking my pills because I was away, I was not comfortable now with my boyfriend… I was then lucky again because he influenced me… he kept asking, ‘are you still picking up your pills?’. I then told him that I missed taking my pills… he encouraged me saying ‘no man please go back there and pick up your pills because we don't know what could happen to both of us or what I can do as a boyfriend, so he influenced me to come back’.” (21 years, Peri‐urban, Two or more consecutive missed visits)

### 3.4 Why do some AGYW never overcome their “PrEP break” or decide to discontinue PrEP?

Those who eventually discontinued PrEP after taking breaks were more likely to mention dosing challenges (i.e. swallowing daily pills) and discouragement from others. They were also less likely to discuss these challenges with study staff or attend study visits. Although some AGYW relocated temporarily, whereby PrEP was not easily accessible, they did not re‐initiate PrEP upon their return.

#### 3.4.1 PrEP access disincentives

When AGYW were asked to describe the dynamics in other AGYW's relationships and how this influenced their PrEP use, many described how the use of PrEP depended on one's relationship status. Such dynamic decision‐making alludes to the adaptive use of PrEP.

“…or this clinic is too far, maybe the person … no longer stays at S. [study area] the person has relocated … or maybe the person decides not to date, maybe the person is not in a relationship, therefore, the person quits PrEP…” (24 years, Peri‐urban, Discontinued PrEP)

“When someone finds someone she thinks she is safe with…she would want to stop taking PrEP…” (18 years, Peri‐urban, Discontinued PrEP)

“I was at school; I was not able to come back here [to the study site]…so I ended up not taking it… I saw that maybe I don't need it that much because at the moment I am not dating anyone…so, I think the day I would be so sure that I have someone…. I will take PrEP if I see a need again to take it‐I will take it.” (18 years, Peri‐urban, Discontinued PrEP)

AGYW who discontinued PrEP also discussed how the lack of familial or peer support may influence one's PrEP‐taking behaviours:

“They [family] might not see it [PrEP] as you are protecting yourself. They might see it as you are being reckless, and you are careless and other things. It is one thing to not being trusted for the things that you do; you might just stop taking it cause you have that thing that either way no one care of this thing I am doing even though it is right…” (18 years, Peri‐urban, Discontinued PrEP)

Partner mistrust and disapproval may cause AGYW to prioritize her relationship over her prevention behaviours, resulting in a disruption to or stopping of PrEP use:

“P:… they [AGYW] have been put in a situation where you have to decide if you take PrEP or… continue to take PrEP our relationship ends, or you choose your relationship over PrEP.”

‘‘I: Ok, so, do you think that changes in relationships can cause these young women to stop taking PrEP?

P: In a way… in a relationship some guys are very much, have power in a way, of dictating [to] you what you should do and where to be, how to [or] who to socialize with and all those things…’’ (24 years, Peri‐urban, Discontinued PrEP)

Of note, only a few participants gave changes in relationships or mistrust as reasons for why they stopped taking PrEP. Ultimately participants felt that AGYW would discontinue PrEP for the following reasons:

“…well according to my experience, 1) it's the side effects and 2) it is not getting maybe support that you thought you'd be getting from people close to you. And 3) the actual reason why you are taking prep cause I believe that the reason why you are taking it should drive you…”. (18 years, Peri‐urban, Discontinued PrEP)

#### 3.4.2 Pill aversion and dosing challenges

Other AGYW specifically spoke about side effects (i.e. nausea, lack of appetite, drowsiness and cramps) or dosing challenges (i.e. not liking pills or struggling to swallow them) as significantly influencing their decision to stop taking PrEP:

“It made me sick. I would take it and I would be nauseous the whole day and be like someone who is pregnant and be sensitive to certain smells… and not have appetite and stuff… I decided that ‘no, it is too much, I cannot handle this’.” (18 years, Peri‐urban, Discontinued PrEP)

Additionally, this participant discussed how PrEP made her lethargic and affected her daily routine. In addition to side effects and dosing concerns, one participant expressed concern about the long‐term impact of PrEP and the potential repercussions if one stops. She also encountered mistrust and discouragement from others:

“My mom and the friends I use[d] to come here with… we did not really trust. Firstly, I was scared of that pill. Then I was concerned about the effects it will have when I have stopped taking it, maybe I'd get sick or something… The people I was talking with told me to stop taking it” (18 years, Rural, Discontinued PrEP)

## 4 DISCUSSION

We sought to understand factors that continue to influence AGYW's PrEP use in the context of a behavioural intervention aimed at maximizing the prevention‐effective use of PrEP. We focused on what influences prolonged breaks in PrEP use and one's decision to re‐initiate or discontinue using PrEP. Little knowledge about PrEP [48], unwelcoming clinical experiences [49, 50], relationship changes and increased perceived risk such as having multiple partners did influence PrEP use behaviours among AGYW interviewed [27, 32, 33, 51–53]. Although similar findings have been previously reported, we further identified key factors that negatively influence PrEP‐use habit formation and PrEP dosing challenges for AGYW in South Africa [54].

Repetitive daily dosing depends on a stable context and consistent cues for habit formation, as this reinforces the mental cue−behaviour association [55]. Despite action planning and skills development to establish a PrEP dosing routine, AGYW discussed forgetting to take their PrEP and waning motivation after PrEP initiation. In this context, AGYW also described constant disruptions to their regular life routines as reasons for numerous unintentional PrEP breaks, as found during temporary relocations, school holidays or visiting extended family/friends. Young women who experience constant disruptions to their regular routines lack consistent contextual cues critical for habit formation [56, 57]. Given the numerous unintentional PrEP breaks experienced by AGYW in our study, PrEP dosing habits were likely never well‐established during critical periods. AGYW also described experiencing behaviour scrutiny and partner mistrust around their PrEP use, both of which can be particularly disruptive when just starting to take PrEP [12, 36]. This further jeopardized the ability to establish a PrEP‐taking routine and revealed the conflicting duality of risk and approval that may encourage AGYW to prioritize their relationships over their HIV prevention needs [26, 27, 36].

Together, routine disruptions and behaviour scrutiny from family, partners and friends, as well as HIV and sexual behaviour‐related stigma, negatively influenced AGYW self‐agency and ability to establish the habits needed for prevention‐effective use of PrEP [23, 27, 36, 52]. Our work reveals the crucial role of supportive social networks for sustained PrEP use, including encouragement and pill reminders [37]. Given the importance of social acceptance among AGYW, peer and family‐based initiatives could prove critical for AGYW's decision to re‐initiate PrEP following breaks in use [11, 21, 36, 52]. Furthermore, strategies that support and/or sustain habit formation should be integrated into adherence support counselling.

### 4.1 Strengths and limitations

A strength of this study was the inclusion of multiple interview categories and study endpoints (Supporting Information, Table S1). These diverse narratives allowed us to understand the social and situational complexities that influence AGYW's PrEP dosing behaviours. Furthermore, the study engaged AGYW from both peri‐urban and rural communities in the Eastern Cape, a high HIV‐burdened province, seldom, if ever, included in research aimed at understanding PrEP uptake and use by AGYW. For the larger CPS study, adherence was measured using TFV‐DP concentrations in DBS samples. However, this adherence measurement was not used for interview participant selection. Consequently, we relied on study attendance/medication refill lists to identify eligible participants and used self‐reported adherence behaviours. Future opportunities to assess near‐real‐time PrEP adherence to inform participant selection could include point‐of‐care urine tenofovir testing [55]. Nevertheless, our IDIs help us understand when and why breaks are taken from PrEP and can inform the design of future PrEP dissemination and intervention support.

## 5 CONCLUSIONS

Daily oral PrEP remains the only available HIV PrEP formulation in South Africa and in much of the world. Consequently, identifying factors that influence the prevention‐effective use of daily oral PrEP, and when and why AGYW take PrEP breaks from it is crucial to understanding how to better support and integrate daily oral PrEP into their lives. Habit formation is a key component for the prevention‐effective use of daily oral PrEP. Disruptions to PrEP dosing, including local travel, behavioural scrutiny, forgetfulness, holiday routines and pick‐up barriers, can deprioritize PrEP, further impacting dosing habit formation [11, 56–59]. Long‐acting oral and injectable PrEP formulations are likely to help alleviate a number of adherence barriers faced by AGYW. Furthermore, expanding the availability of PrEP options in South Africa may also address logistical barriers related to access. Though there are initiatives to expand long‐acting formulations that should not be neglected, there is severely limited access to long‐acting PrEP formulations in South Africa and globally. Given the strong likelihood that oral PrEP will remain “the only game in town” in many parts of the world for some time, much more needs to be done to ensure that AGYW, and all individuals at‐risk for HIV, are able to access and use daily oral PrEP in a prevention‐effective manner [60, 61].

## COMPETING INTERESTS

The authors declare that they have no competing interests.

## AUTHORS’ CONTRIBUTIONS

AM‐M and L‐GB conceptualized the main Community PrEP Study and acquired funding. SH and CC contributed to the NIMH grant proposal development and submission, and analytic support. AM‐M and JD conceptualized complementary study activities and acquired additional funding. AM‐M, L‐GB, LF, MA and MM contributed to the qualitative design and development of interview guides. Data coding, analysis and interpretation were conducted by LDV, EKM, LF, MA and JD. LDV drafted the initial manuscript under the guidance of EKM, LF, MA and JD, through weekly team meetings and revised critically by AM‐M. All authors contributed to the writing, review and approval of the manuscript.

## FUNDING

The Community PrEP study (ClinicalTrials.gov NCT03977181) was funded by the National Institute of Mental Health (NIMH) of the U.S. National Institutes of Health (award number R01MH114648 to AM‐M and L‐GB). Complementary funding by The Bill and Melinda Gates Foundation via the South African National HIV Think Tank (provided to AM‐M and JD). The study was partially supported by NIAID funding/PUMA (R01AI143340 provided to MG). This study was conducted with the approval of the Eastern Cape Provincial and Buffalo City Metro District Health Departments and the University of Cape Town Human's Research Ethics Committee (Ref no.: 289/2018).

## Supporting information


**Table S1**. In‐depth interview categories.
Interview categories that explore PrEP use experiences, including missed adherence visits, discontinued use, unique patterns of medication use and defined CPS study endpoints (e.g. seroconverted, study arm‐experience interviews).

## Data Availability

The data that support the findings of this study are available on request from the corresponding author. The data are not publicly available due to privacy or ethical restrictions.
