# Supplementary material for: Factors that influence adolescent girls and young women's re‐initiation or complete discontinuation from daily oral PrEP use: a qualitative study from Eastern Cape Province, South Africa
Source: J Int AIDS Soc. 2023 Sep 27;26(9):e26175. doi: 10.1002/jia2.26175 (PMC10533377; doi:10.1002/jia2.26175)
Supplement: Supplementary file 1 — Table S1. In‐depth interview categories. Interview categories that explore PrEP use experiences, including missed adherence visits, discontinued use, unique patterns of medication use and defined CPS study endpoints (e.g. seroconverted, study arm‐experience interviews). [file JIA2-26-e26175-s001.docx]

| **Table 1. In-depth Interview Categories**  *Interview categories that explore PrEP use experiences including missed adherence visits, discontinued use, unique patterns of medication use, and defined CPS study endpoints (e.g., seroconverted, study arm-experience interviews)* | | | |
| --- | --- | --- | --- |
| **IDI category** | **Description** | **IDI Key Focus Areas** | **Interviews N=53** |
| **Two or more consecutive missed visits** | Participants who missed two or more consecutive monthly medication refills during the 2-year follow-up period | Participant risk behaviors (including perceived risk), reasons for missed pick-up (for self and others), reasons for re-initiation, decisions for PrEP use, and seasons of risk | 17 |
| **Discontinued PrEP** | Participants who decided to discontinue/stop PrEP during the 2-year follow-up period | Social network influences, PrEP accessibility, and preferred platforms, study experiences, general and personal reasons for discontinued use, support recommendations for continued use | 7 |
| **Unique pattern of medication use** | Participants who show high participation in monthly study sessions yet have low drug adherence levels (confirmed by DBS). Participants suspected of stockpiling, sharing, or discarding PrEP were identified by adherence counselors or study nurses. | Changes in motivation, PrEP routine, and storage, non-PrEP related reasons for study attendance, general and personal reasons for sharing, stockpiling, discarding PrEP, recommendations for adherence support | 14 |
| **Seroconverts** | Participants who tested HIV positive during the study. HIV status was confirmed by a rapid finger-prick HIV test as per South African national guidelines confirmed with an ELISA test. | Testing and referral experiences, participant’s risk perception, sexual behavior changes during PrEP, identification, and influence of key supporters, patterns of use, and perceived needs | 4 |
| **Serial Interviews M18-M24** | Participants who participated in two individual in-depth interviews: one at month 2 and one between month 18 and 24 study follow-up | Motivation for continued PrEP use, barriers, and facilitators for PrEP, study arm experiences, the influence of PrEP on sexual behavior, recommendations for future roll-out | 4 |
| **Study Arm Experiences** | Participants who show high participation in monthly study sessions were interviewed by 15-24 months about their study arm experiences and PrEP use  • Medication Pick-up (Control arm)  • One-on-one  • Health Club | PrEP knowledge and prior use, PrEP routine, and storage recollecting adherence support arm study details, group member versus one-on-one study experiences, study evaluation including activities, and preferences | 7 |
